# One-Carbon Metabolism Regulates Embryonic Stem Cell Fate Through Epigenetic DNA and Histone Modifications: Implications for Transgenerational Metabolic Disorders in Adults

**DOI:** 10.3389/fcell.2019.00300

**Published:** 2019-11-21

**Authors:** Lon J. Van Winkle, Rebecca Ryznar

**Affiliations:** ^1^Department of Biochemistry, Midwestern University, Downers Grove, IL, United States; ^2^Department of Medical Humanities, Rocky Vista University, Parker, CO, United States; ^3^Molecular Biology, Department of Biomedical Sciences, Rocky Vista University, Parker, CO, United States

**Keywords:** one-carbon metabolism, folate, threonine metabolism, methionine metabolism, inner cell mass, embryonic stem cells, epigenetic histone modification, metabolic syndrome

## Abstract

Human (h) and mouse (m) embryonic stem (ES) cells need specific amino acids to proliferate. mES cells require threonine (Thr) metabolism for epigenetic histone modifications. Thr is converted to glycine and acetyl CoA, and the glycine is metabolized specifically to regulate trimethylation of lysine (Lys) residue 4 in histone H3 (H3K4me3). DNA methylation and methylation of other H3 Lys residues remain unimpaired by Thr deprivation in mES cell culture medium. Similarly, hES cells require methionine (Met) to maintain the Met-SAM (S-adenosyl methionine) cycle of 1-carbon metabolism also for H3K4me3 formation. H3K4me3 is needed specifically to regulate and maintain both mES and hES cell proliferation and their pluripotent states. Better understanding of this regulation is essential since treatment of human diseases and disorders will increasingly involve hES cells. Furthermore, since ES cells are derived from their progenitor cells in preimplantation blastocysts, they serve as models of 1-carbon metabolism in these precursors of all mammalian tissues and organs. One-carbon metabolism challenges, such as a maternal low protein diet (LPD) during preimplantation blastocyst development, contribute to development of metabolic syndrome and related abnormalities in adults. These 1-carbon metabolism challenges result in altered epigenetic DNA and histone modifications in ES progenitor cells and the tissues and organs to which they develop. Moreover, the modified histones could have extracellular as well as intracellular effects, since histones are secreted in uterine fluid and influence early embryo development. Hence, the mechanisms and transgenerational implications of these altered epigenetic DNA and histone modifications warrant concerted further study.

## Introduction

Both sperm and egg cells contain DNA and histones that contribute to the genetic and epigenetic complements of mammalian conceptuses. The epigenetic complements of these gametes and early embryos are altered by environmental challenges to their one-carbon amino acid metabolism (Fleming et al., 2018; Clare et al., 2019; Goyal et al., 2019; Velazquez et al., 2019). Such challenges increase the prevalence of metabolic syndrome and related disorders in offspring that develop from the embryonic stem (ES) progenitor cells in these early conceptuses (Van Winkle and Ryznar, 2018).

Within days of conception, embryos give rise to ES progenitor cells and another type of cell in preimplantation blastocysts. A trophectoderm, one cell thick, forms the surface of blastocysts. This trophectoderm attaches to uterine epithelial cells to initiate implantation. Pluripotent cells, termed the inner cell mass (ICM), reside within the trophectoderm layer. After implantation, cells in the ICM differentiate into progenitors of all tissues in the offspring. Thus, environmentally induced epigenetic changes in ICM cells result in such changes in adult tissues and organs (Clare et al., 2019; Velazquez et al., 2019). Since the ICM is used to produce pluripotent ES cell lines, ES cells serve as an experimental model for environmental challenges to their progenitor cells in the ICM of blastocysts.

## Mouse Embryonic Stem (mEs) Cells Require Threonine (Thr) Metabolism to Remain Pluripotent

### mES Cells Require Thr One-Carbon Metabolism for Specific, Epigenetic Histone Methylations

Mouse ES cells do not proliferate and begin to differentiate if Thr is not supplied in their culture medium (Wang et al., 2009). These cells catabolize Thr to glycine and acetyl CoA, which are used for and help to regulate epigenetic methylations and acetylations in mES cells (Jog et al., 2017). Di- and tri-methylation of lysine (Lys) residue 4 in histone H3 (H3K4me3) slows dramatically and selectively when Thr is not supplied in the medium, while methylation of DNA and other H3 Lys residues continues normally (Shyh-Chang et al., 2013). Mouse ES cells need H3K4me3 to proliferate and remain pluripotent (Ang et al., 2011; Kilberg et al., 2016; Chen and Wang, 2019). Hence, Thr, taken up from the culture medium by mES cells, seems somehow to be metabolized selectively to provide 1-carbon units for H3K4 methylation.

### Might Membrane Transporters Feed Amino Acids to Specific Intracellular Metabolic Compartments?

In the preimplantation blastocyst, leucine taken up by the trophectoderm triggers mammalian target of rapamycin (mTOR) signaling, trophoblast motility, and penetration of the uterine epithelium (Van Winkle et al., 2006). This signaling requires leucine uptake via the B^0,+^ amino acid transporter. Leucine uptake by other transporters does not result in trophoblast motility. B^0,+^ either selectively directs leucine to sites of mTOR signaling, or it generates other signals needed to synergize with mTOR (González et al., 2012).

**FIGURE 1:**
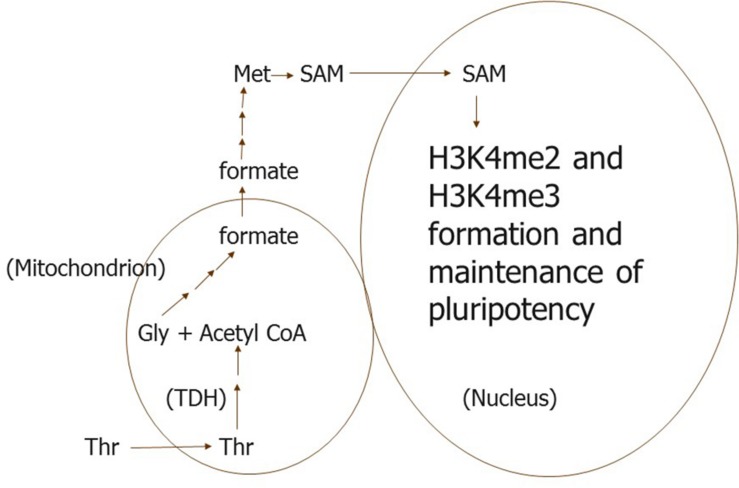
We propose that a subpopulation of perinuclear mitochondria is specialized to take up only Thr and metabolize it to formate in *mouse* ES and their progenitor cells. The formate is then converted to 1-carbon units in a pool of SAM used specifically for H3K4me di- and tri-methylation. TDH, Thr dehydrogenase.

In mES cells, selective use of Thr for H3K4 methylation may be more complex. Since Thr metabolism begins in perinuclear mitochondria in mES cells (Wanet et al., 2015; Lees et al., 2017; Woods, 2017), we propose that Thr is taken up selectively by a subpopulation of these organelles (Figure 1). Thr is then metabolized to formate, which leaves the mitochondria, and is converted to S-adenosyl methionine (SAM) methyl groups needed in the nucleus for H3K4 methylation (Scotti et al., 2013; Harvey, 2019). The formate from this subpopulation of mitochondria must be directed somehow to sites of H3K4 methylation in the nucleus, since only 1-carbon metabolism of Thr can be used to methylate this H3 residue (Shyh-Chang et al., 2013). Other 1-carbon donors cannot be used for this purpose unless supplied in excess to mES cells.

Alternatively, Thr could be directed to the pertinent subpopulation of perinuclear mitochondria by one of at least three plasma membrane Thr transport proteins (Formisano and Van Winkle, 2016). One of these transporters could conceivably direct Thr to the subpopulation of perinuclear mitochondria much like the B^0,+^ transporter might selectively directs leucine to the site of mTOR signaling in the trophectoderm.

### Does Thr Also Initiate Signaling by Interacting Directly With One of Its Transporters in Lipid Rafts?

In addition to providing metabolites for epigenetic DNA and histone methylation and acetylation (Jog et al., 2017), Thr itself is likely needed for proliferation and maintenance of an undifferentiated state in mES cells (Ryu and Han, 2011). Thr promotes expression of cMyc through processes mediated by mTOR in these cells. Intact lipid rafts containing a Thr transporter seems essential for this signaling, since the signaling cannot occur in mES cells when these rafts are disrupted. Either an intact raft supports Thr transport and resultant intracellular signaling by the amino acid itself, or raft caveolae initiate signaling during Thr interaction with its transporter in the plasma membrane. As an alternative to a present thesis (e.g., Figure 1), perhaps the latter signaling and Thr catabolism together foster H3K4 methylation by somehow selectively activating pertinent histone modifying enzyme(s).

By interfering with Thr transport (Van Winkle et al., 2014; Formisano and Van Winkle, 2016) as well as catabolism (Wang et al., 2009), the Thr analog, 3-hydroxynorvaline (3-HNV), inhibits proliferation of mES cells. Similarly, this Thr transport and resultant signaling as well as catabolism in the ICM of blastocysts would help maintain pluripotency in these mES progenitor cells.

## Human Embryonic Stem (hEs) Cells Need Methionine (Met) Metabolism for Proliferation

### Met Metabolism for Specific, Epigenetic Histone Methylations May Also Be Partially Compartmentalized in hES Cells

Naïve and primed mES cells are epigenetically different, and hES cells seem to better correspond to primed mES cells (Takahashi et al., 2018). Nevertheless, 1-carbon metabolism regulates hES cell proliferation. Met is required by hES cells, and their progenitors in the inner cell masses of blastocysts, for specific, epigenetic histone methylations. If Met is not in the culture medium, hES cells do not proliferate but undergo apoptosis (Shiraki et al., 2014). Exogenously supplied Met is metabolized to SAM in hES cells; these cells utilize SAM to carry out epigenetic methylations. A lack of Met (or supplemental SAM) in the medium nearly eliminates di- and tri-methylation of Lys residue 4 in histone H3 (H3K4me3). Global DNA methylation is also decreased by a modest amount through Met deprivation of hESC cells (Shiraki et al., 2014), but methylation of other H3 Lys residues remains unimpaired (Kilberg et al., 2016). As for mES cells, H3K4me3 maintains hES cell proliferation and pluripotency.

**FIGURE 2:**
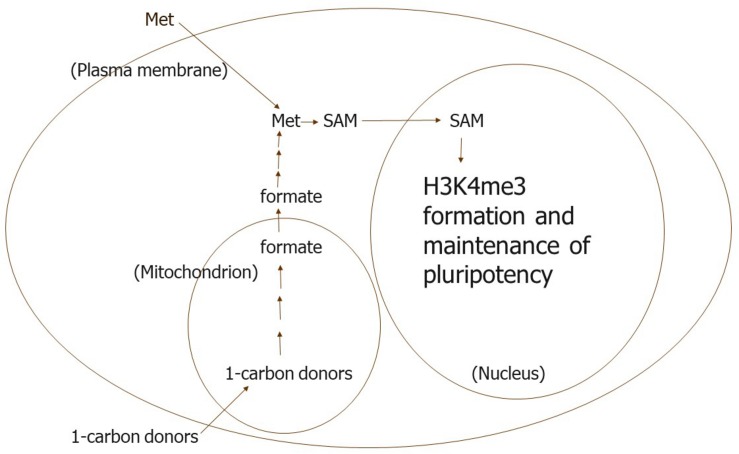
We propose that Met is needed in the culture medium to maintain the intracellular concentrations of metabolites in the Met-SAM methylation cycle that are needed for H3K4me3 formation in *human* ES and their progenitor cells. In our view, other methyl transferases are able to use SAM preferentially when its intracellular concentration is lower possibly because the *K*_*m*_ values of these other transferases are lower than the transferases that methylate H3K4.

Met is needed in the culture medium apparently to maintain adequate concentrations of metabolites in the Met-SAM methylation cycle in hES cells (Figure 2). This cycle seems to be used preferentially by methyltransferases other than those catalyzing methylation of H3K4 when the concentration of metabolites in the cycle are low. Moreover, Met might be easily depleted from hES cells when not present in the culture medium owing to exchange of Met for other bulky zwitterionic amino acids in the medium. The only good transporter of Met in hES cell membranes appears to be one or more of those corresponding to the Na^+^-independent system L, which is used for such exchange (Van Winkle and Ryznar, 2019).

### An Enigma: Thr Recuses hES Cells From Growth Inhibition by the Thr Analog, 3-HNV, but Not Because Thr Is Needed for One-Carbon Metabolism or Protein Synthesis

According to Wang and associates Wang et al. (2009), the Thr analog, 3-HNV, inhibits mES cell proliferation because it inhibits Thr dehydrogenase (TDH) and metabolism of Thr to glycine and acetyl CoA. We found, however, that 3-HNV also inhibits hES cell proliferation (Van Winkle et al., 2014). Unlike mouse cells, hES cells lack functional TDH and so do not catabolize Thr to the 1-carbon units needed for epigenetic H3K4 methylation and maintenance of pluripotency. Somewhat surprisingly, 3-HNV inhibits hES cell proliferation in a manner morphologically similar to the way it arrests mES cell growth (Van Winkle et al., 2014).

It has been proposed that 3-HNV inhibits proliferation of cells lacking TDH activity through 3-HNV incorporation into protein in place of Thr (Najafzadeh, 2018). But 3-HNV does not inhibit proliferation of other mouse cell lines or HeLa cells (a human cell line; Wang et al., 2009), so it is unlikely to inhibit hES cell growth owing to its incorporation into protein. An excess of Thr in the culture medium does, however, rescue hES proliferation from 3-HNV inhibition. These findings support the theory that Thr fosters hES and mES cell proliferation through signaling, such as that requiring intact lipid rafts, that is separate from (and in addition to) regulation of epigenetic DNA and histone modifications by 1-carbon metabolites of Thr in mES cells (Van Winkle et al., 2014).

## Methyl Donor Perturbations, Including a Maternal Low Protein Diet, Likely Alter Transgenerational Epigenetic Dna and Histone Modifications in mEs Progenitor Cells

### Does a More Invasive and Pinocytic Trophectoderm Supply More Thr to mES Progenitor Cells and Modify Placentation?

When female mice consume a LPD during preimplantation development of their conceptuses, trophoblast cells adapt in several ways in blastocysts. The trophectoderm exhibits more proliferation, motility during penetration of the uterine epithelium, and endocytic sequestration of uterine fluid proteins (reviewed by Fleming et al., 2018). These proteins are likely hydrolyzed to supply Thr to mES progenitor cells (Van Winkle and Ryznar, 2019), so increased accumulation of the proteins should result in delivery of more Thr to these pluripotent cells. More protein hydrolysis could also supply more leucine and arginine and, thus, augment trophoblast motility (González et al., 2012).

Other changes may also cause mES progenitor cells to accumulate more Thr. Mouse ES cells take up Thr via amino acid exchange (Formisano and Van Winkle, 2016). Thus, Thr can be accumulated against its chemical potential gradient only if other amino acids are first taken up against their gradients. For instance, mES cells should be able to accumulate alanine against its gradient via the Na^+^-dependent amino acid transport system A (Van Winkle and Ryznar, 2019). This alanine could then exit the cells via system ASC and drive uptake of Thr into mES cells. In this regard, alanine may accumulate in the environment of mES progenitor cells when their mothers are consuming a LPD. When blastocysts develop from the 2-cell stage in culture medium without added amino acids, their alanine concentration is six times higher than in morphologically similar blastocysts developing *in vivo* (Van Winkle and Dickinson, 1995). While this *in vitro* culture system is an extreme model, it might mimic the effects of LPD well enough to indicate how mES progenitor cells could accumulate more Thr in exchange for alanine. Greater Thr accumulation could alter the supply of 1-carbon units to SAM and its regulation of H3K4me di- and tri-methylation.

Maternal consumption of a LPD during the pre- and peri-implantation stages of development also results in development of more efficient placentas (Fleming et al., 2018). More vigorous trophoblast invasion of the uterine epithelium owing to maternal LPD consumption likely leads to this development. Likewise, greater delivery of nutrients by the yolk sac placenta occurs, owing to altered epigenetic histone modifications in the primitive endoderm that gives rise to this placenta (Fleming et al., 2018). In addition, switching from LPD to control diet consumption by pregnant mice at the time of blastocyst implantation causes the somatic cell lineages of mES progenitor cells to decrease then raise, respectively, the level of some of their epigenetic DNA modifications relative to the control rate. Such changes in the nutrient supply to conceptuses also likely change epigenetic histone modifications during early embryo development.

### Altered Epigenetic Histone Modifications in mES Progenitor Cells Likely Persist in Future Generations Without Further Methyl Donor Perturbations

Dysfunction of endothelial cells and elevated blood pressure occur in grandprogeny of rat dams consuming a LPD during pregnancy, in the absence of protein restriction of the F_1_ generation, probably owing to transgenerational epigenetic DNA and histone modifications (Torrens et al., 2008). For the same reasons, impaired placental function or older age of F_0_ mothers result in transmission of cardiorenal and metabolic alterations to their grandprogeny (Gallo et al., 2012; Master et al., 2015). Moreover, extracellular as well as intracellular actions of histones could have occurred, owing to uterine secretion of the modified histones by F_1_ females (Van Winkle and Ryznar, 2018).

Epigenetic methyl donor challenges can also be genetic. For example, impairment of folate metabolism through genetic mutation in F_0_ mice results in suppression of epigenetic methylation, with transgenerational effects on development of wild-type progeny mice, for at least five generations (Padmanabhan et al., 2013). Epigenetic modifications in the germline of F_1_ females cause congenital malformations, whereas alterations in the uterine environment of F_1_ mothers produce growth defects. The altered uterine environment likely includes epigenetically modified histones, since uterine cells of F_1_ wild-type mice exhibited hypomethylation of DNA and probably histones, and uterine fluid contains histones that likely influence early embryo development (Van Winkle, 2017; Van Winkle and Ryznar, 2018). Histones act both intracellularly and extracellularly, however, so the effects of their hypomethylation probably include both congenital malformations and growth defects.

The proposed actions of extracellular histones are also illuminated by methyl donor supplementation that prevents transgenerational obesity amplification in F_3_ agouti viable yellow mice (Waterland et al., 2008). In this study, germ cells of F_0_ mice were formed in the unsupplemented control environment since their parents received no methyl donor supplementation. As a result, mES progenitor cells that produced F_1_ mice had the epigenetic modifications associated with the control diet, so the organs that developed in F_1_ mice did not benefit from methyl donor supplementation. We propose that the resultant uterine secretion of altered histones by F_1_ mice (Van Winkle and Ryznar, 2018) contributed to development of obesity in the F_2_ generation. In contrast, methyl donor supplementation of F_0_ mice improved development of F_1_ germ cells, so organs, such as the uterus, also developed more normally in the F_2_ offspring of F_1_ mice. As a consequence, transgenerational obesity amplification was not observed in F_3_ mice, owing to methyl donor supplementation beginning in the F_0_ generation (Waterland et al., 2008).

### Other Sources of Extracellular Histones in the Reproductive Tract

In addition to histones secreted in uterine fluid (Van Winkle, 2017; Van Winkle and Ryznar, 2018), other sources of extracellular histones include seminal fluid and dead sperm cells (Fung et al., 2004; Miller et al., 2010; Miller, 2018). Paternal as well as maternal environmental challenges adversely affect peri-implantation development of embryos through intergenerational epigenetic modifications (Clare et al., 2019; Su and Patti, 2019). These changes are likely present in extracellular and intracellular histones, thus providing one mechanism by which paternal diet could program offspring health through both seminal plasma- and sperm-specific pathways (Watkins et al., 2018).

### Perturbations in Methyl Donor Supply Also Likely Alter Epigenetic DNA and Histone Modifications in Pre- and Peri-Implantation *Human* ES Progenitor Cells

Epigenetic DNA methylation marks change during formation of early embryos, and these changes foster normal tissue development. Human oocytes have high levels of the folate receptor FOLR1, suggesting that folate uptake for 1-carbom metabolism is important during the maternal to zygotic transition (Strandgaard et al., 2017). Babies born after IVF procedures have higher rates of disorders related to imprinting and epigenetic changes, maybe because commercial IVF media does not contain intermediates of 1-carbon metabolism, such as folates and methionine (Menezo et al., 2019). Moreover, epigenetic changes occurring during the activation of induced pluripotent stem cells require an optimal SAM pool for appropriate reprograming back to the pluripotent state (Fernández-Arroyo et al., 2015). This reprograming can be influenced by nutritional deficiencies of needed cofactors derived from vitamin B complexes. The altered methyl donor metabolism affects DNA methylation, histone modifications, and miRNAs. In addition, methyl donor challenges in humans, such as a LPD, alters epigenetically programed ES cell progenitor differentiation (Wu et al., 2019).

Furthermore, the differentially methylated CpG islands that regulate genomic imprinting may be highly susceptible to changes in diet during pre- and peri-implantation development. With over 150 imprinted genes identified in the human genome, and many human diseases associate with dysregulation of these areas, changes in diet affecting these regions could have profound effects on the offspring. For example, folic acid supplementation before versus during pregnancy showed changes in DNA methylation patterns specifically at the imprinted H19/IGF2 locus in infants. The authors proposed that since methylation marks regulating imprinted genes are acquired at differentially methylated DNA regions before gastrulation, their methylation patterns may reveal environmental exposures such as a change in diet or toxin exposure (Hoyo et al., 2011).

Other locations within the human genome may be affected by changes in epigenetic marks as the result of diet fluctuations. For example, active H3K4me3 and repressive H3K27me3 maintain a pluripotent epigenetic state in ES and their progenitor cells (Wu et al., 2019). In hES cells, there are also abundant non-CpG methylated cytosines that may be important to early development (Lister et al., 2009).

A maternal LPD also predisposition human as well as rodent offspring to metabolic syndromes by modifying genes including H19, IGF2, UBE3A, POMC, GR, and PPAR-α (Gong et al., 2010; Lillycrop and Burdge, 2011). These modified genes are related to imprinting, metabolism, or the stress response. Such changes in methylation patterns of the genome in the early embryo, after birth, or early in life provide evidence of how environment contributes to transgenerational human disease states.

## Conclusion

Epigenetic DNA and histone modifications in ES and their progenitor cells in early embryos require membrane transport and compartmentalized metabolism of amino acids. One-carbon metabolites produced specifically to regulate H3 methylation in mES and hES cells require Thr and Met from the culture medium. ES cells require these 1-carbon units to produce H3K4me3 and remain undifferentiated. Further understanding of this regulation is essential. All adult tissues and organs arise from ES progenitor cells, and treatment of human diseases and disorders will increasingly involve hES cells.

Furthermore, metabolic syndrome and related disorders often result from altered 1-carbon metabolism in ES progenitor cells. This altered 1-carbon metabolism produces modified epigenetic DNA and histone methylations. Such epigenetically produced diseases and disorders can become transgenerational phenotypes in humans. Extracellular as well as intracellular histones may alter development beginning in early embryos when these histones are produced, through perturbation of 1-carbon methyl donor metabolism, and secreted in uterine fluid.

## Author Contributions

Both authors created sections of the manuscript, reviewed and provided feedback to each other that facilitated its critical revision, and approved the final version of the manuscript for publication.

## Conflict of Interest

The authors declare that the research was conducted in the absence of any commercial or financial relationships that could be construed as a potential conflict of interest.
